# Detection of JC Virus-Specific Immune Responses in a Novel Humanized Mouse Model

**DOI:** 10.1371/journal.pone.0064313

**Published:** 2013-05-20

**Authors:** Chen Sabrina Tan, Thomas A. Broge, Edward Seung, Vlad Vrbanac, Raphael Viscidi, Jennifer Gordon, Andrew M. Tager, Igor J. Koralnik

**Affiliations:** 1 Division of Infectious Diseases, Beth Israel Deaconess Medical Center, Boston, Massachusetts, United States of America; 2 Center for Virology and Vaccine Research, Department of Medicine, Beth Israel Deaconess Medical Center, Boston, Massachusetts, United States of America; 3 Division of NeuroVirology, Department of Neurology, Beth Israel Deaconess Medical Center, Boston, Massachusetts, United States of America; 4 Center for Immunology and Inflammatory Diseases, Division of Rheumatology, Allergy and Immunology, Massachusetts General Hospital, Charlestown, Massachusetts, United States of America; 5 Johns Hopkins Medical Center, Baltimore, Maryland, United States of America; 6 Department of Neuroscience, Center for Neurovirology, Temple University School of Medicine, Philadelphia, Pennsylvania, United States of America; 7 Harvard Medical School, Boston, Massachusetts, United States of America; University of Utah School of Medicine, United States of America

## Abstract

Progressive Multifocal Leukoencephalopathy (PML) is an often fatal disease caused by the reactivation of the JC virus (JCV). Better understanding of viral-host interactions has been hampered by the lack of an animal model. Engrafting NOD/SCID/IL-2-Rg (null) mice with human lymphocytes and thymus, we generated a novel animal model for JCV infection. Mice were inoculated with either a PML isolate, JCV Mad-4, or with JCV CY, found in the kidney and urine of healthy individuals. While mice remained asymptomatic following inoculation, JCV DNA was occasionally detected in both the blood and the urine compartments. Mice generated both humoral and cellular immune responses against JCV. Expressions of immune exhaustion marker, PD-1, on lymphocytes were consistent with response to infection. Using this model we present the first *in vivo* demonstration of virological and immunological differences between JCV Mad-4 and CY. This model may prove valuable for studying JCV host immune responses.

## Introduction

Progressive multifocal leukoencephalopathy (PML) is a rare yet often fatal disease of the brain for which there is no available treatment [1]. PML results from lytic destruction of oligodendrocytes by JC virus (JCV). While up to 80% of healthy individuals are seropositive for JCV [2], PML occurs in immunosuppressed individuals, including those with HIV, malignancies, transplant recipients, and individuals treated with immunomodulatory medications. Asymptomatic primary infection of JCV occurs in childhood and the virus remains detectable in the urine of one third of healthy individuals without causing any disease [3]. In patients with PML, active JC viral replication in the brain results in lysis of oligodendrocytes and consequently demyelination. Although prognosis is poor for patients with PML, our studies have demonstrated better survival by those patients with detectable host cellular immune responses against JCV [4], [5], [6]. However, JCV-specific T cell responses are low in fresh blood samples, requiring *in vitro* stimulation with viral antigen to obtain robust results [7], [8]. Better understanding of host immune responses and JCV pathogenesis is crucial for developing anti-viral treatments. Therefore, it is extremely important to develop an animal model for studying JCV interactions with the immune system. Unfortunately, JCV, similar to other polyomaviruses, is highly species-specific and active replication is only permissive in the human host. Recently, mice engrafted with human fetal stem cells and thymus, have been employed in the study of other species-specific viruses [9]. Specifically, the immunodeficient mice, NOD-SCID/IL-2Rg (null) or NSG, are transplanted with human fetal bone marrow, liver and thymus (BLT) after sublethal dose of irradation. After reconstitution with human immune cells, these mice can generate a full spectrum of human cells including T cells, B cells, NK cells, macrophages, and dendritic cells. The persistent residual mouse lymphocytes generally make up less than 5% of total lymphocytes. Studies have demonstrated immune functions of these human cells against human-specific viruses including HIV and EBV [10], [11], [12]. The prospect of using this humanized mouse model to study JCV immune response is further enhanced by the fact that in addition to kidney tubular epithelial cells, the bone marrow is a site of latency and reactivation for JCV [13]. Therefore, we hypothesize that the engrafted human hematopoietic cells will enable active JCV replication in these mice and model immune response.

JC viral tropism and virulence is determined in part by the non-coding hypervariable regulatory region (RR) [14]. While isolates from urine have a stable non pathogenic RR, known as archetype, viral strains from the brain or CSF of PML patients contain viral isolates mostly with rearranged RR due to deletions and duplications. These were initially isolated at the University of Wisconsin in Madison and were called Mad-type [15]. It has yet to be determined whether archetype or the Mad-type of JC virus causes primary infection in humans. Furthermore, it is not known if different viral strains elicit different host immune responses. We, therefore, determined to compare the infection of brain-derived rearranged isolate, JCV Mad-4, with the urine-derived archetype isolate, JCV CY, in our humanized BLT mouse model.

## Materials and Methods

### Humanized BLT mice

#### Ethics statement

This is study was carried out in accordance with the recommendations in the Guide of the Care and Use of Laboratory Animals of the National Institute of Health. The protocol was approved by the Subcommittee on Research Animal Care of Massachusetts General Hospital (Federal Assurance A3596-01, protocol 2009/N000028/3). All efforts were made to minimize animal suffering.

Immunodeficient mice, NOD-SCID/IL-2Rg(null) or NSG, were reconstituted with HLA A_0201_ –positive human fetal liver CD34^+^ cells and transplanted with autologous fetal thymus and liver as previously described [10].

### JC virus

JCV Mad-4 and JCV CY viral strains were propagated in astrocytic SVG-A and kidney epithelial COS-7 cells, respectively, both of which express the SV40 T-antigen. Cells were transfected with pBJC (Mad-4) and pJC-CY (CY) plasmid DNAs digested to release the entire JCV genome in linear form. Cells were subcultured for approximately 3 to 4 weeks until CPE were observed, then virus was collected from adherent and non-adherent cells by freeze-thawing and treatment with 0.25% deoxycholic acid. Virus-containing supernatant was stored at −80°C until needed. Viral titer was determined by qPCR and HA assay as described previously [16], [17]. Each virus was injected intraperitoneally at a dose of 2,500 HAU (equivalent to 2.5×10^7^ viral copies) or 5,000 HAU (equivalent to 5×10^7^ viral copies) per mouse for both viruses.

### Extraction of viral nucleic acids and detection with quantitative PCR (qPCR)

#### DNA

The Qiagen MinElute kit (Qiagen, CA) was used following the manufacturer's instructions for DNA extraction from urine and plasma. The Qiagen Blood kit was used following the manufacturer's instructions for DNA extraction from whole blood. Because of the variable volume of blood and urine samples obtained from each animal, viral DNA were first extracted from the samples. An aliquot was used for determination of total DNA concentration. The results of JC viral load were expressed per µg of extracted DNA, rather than per ml of the sample. Qiagen Tissue kit was used for DNA extraction of organs. QPCR was used to detect and quantify JCV DNA in all samples as previously described [18]. The standard curve ranges from 1 copy to 10^6^ copies per sample well. Results above 1 copy are considered positive.

#### RNA

Total RNA was extracted using RNeasy Mini kit (Qiagen, CA) and reverse transcribed using RNA-to-cDNA kit (Applied Biosystems). Real-time PCR was performed on complimentary DNA using TaqMan Gene Expression master mix (Applied Biosytems). Reactions were performed in 50 µl in triplicate on a 7300 Real Time PCR system (Applied Biosystems).

### Measuring anti-JCV antibodies in serum

A virus-like particle-based ELISA was used to detect IgM and IgG antibodies to JCV capsid as previously described [19].

### Lymphocyte phenotyping

Direct immunofluorescence staining of whole blood was performed using mouse CD45 (eBiosciences; clone 30-F11), human CD45 (Invitrogen; clone H130), CD3 (BD Bioscience; clone SP34-2), CD4 (Invitrogen; clone S3.5), CD8 (BD Bioscience; clone RPA-T8), CD19 (BD Bioscience; clone SJ25C1), and PD-1 (BioLegend; clone EH12.2H7) for 25 minutes at 4°C. RBC's were lysed by treatment with BD FACS Lysing Solution for 10 minutes at 4°C. Cells were washed twice with PBS containing 2% FBS. Analysis was performed on a LSRII flow cytometer (BD Biosciences) and FlowJo software (Treestar Inc.).

### Generation of human B lymphoblastoid cell line (BLCL)

EBV-transformed autologous B-lymphoblastoid cell line (BLCL) was generated by incubating 2×10^7^ PBMC in B95.8 supernatant, an EBV infected marmoset cell line, at 37°C for 3 hours, resuspended (1×10^6^ cells/ml) in RPMI 1640 containing 20% FBS and 1 µg/ml cyclosporine, and plated in 48-well plates. After 7 days, cells were fed with RPMI 1640 containing 12% FBS every 2–3 days and monitored for signs of immortalization. After immortalization, BLCL were used as feeder cells for the ELISpot assay.

### Isolation of Splenocytes

Spleens from individual mice were removed, placed in RPMI 1640 with 12% FBS, and disrupted by passage through a 70 µm filter. RBCs were lysed by using ACK solution. Cells were washed, counted, and resuspended (3–3.5×10^6^ cells/ml) in RPMI 1640 containing 12% FBS. 7×10^6^ cells per well were cultured in a 12-well plate with each peptide pool. The JCV VP1 peptide pools comprised of 15 amino acids(aa) peptides overlapping by 11-aa and encompassed the entire VP1 protein including amino acid variations in both JCV Mad and CY types of viruses. Additionally, 3.5×10^6^ cells were stimulated with HLA-A*0201-restricted JCV VP1 epitopes, VP1_p36_ or VP1_p100_, for tetramer staining. 72 hours later recombinant human IL-2 (50 U/ml) was added to each sample. After 10 days of in vitro stimulation, splenocytes were used to assess JCV specific cellular immune responses.

### Elispot

Prior to ELISpot assay, BLCL (2×10^6^ cells/ml) were incubated with each peptide pool for 16 hours. After incubation, BLCL were treated with mitomycin C (0.5 mg/ml) for 2.5 hour, washed twice and resuspended at 1×10^6^ cells/ml. After pretreatment, 5×10^4^ BLCL were co-cultured with 5×10^4^ splenocytes on 96-well MultiScreen HTS plates (Millipore) previously coated with anti-human IFN-γ at 5 µg/ml and stimulated with the same peptide pool as used for stimulation at beginning of culture. Each test sample was run in triplicate. After 36 hour incubation at 37°C, plates were washed, incubated with anti-human IFN-γ biotin (Biosource) for 2 hours at room temperature, washed, and incubated with streptavidin (Southern Biotechnology). The Plates were developed with nitroblue tetrazolium-5-bromo-4-chloro-3-indolylphosphate chromogen (Pierce) and analyzed on an Immunospot Analyzer (Cellular Technology Limited). An ELISpot result was considered positive when the numbers of spot forming units (SFU) were greater than three times the baseline value, and when the number of SFU is greater than 50 per 10^6^ cells after subtraction of baseline. Results were reported after subtraction of baseline values.

### Intracellular Cytokine Staining

1×10^6^ splenocytes were incubated in RPMI 1640 with 12% FBS medium alone (unstimulated), with a peptide pool of the VP1 peptide (2 mg/ml), or with PMA and ionomycin (1 µg/ml and 5 µg/ml, respectively) at 37°C for 6 hours. All samples received monensin (GolgiStop; BD Bioscience) after the first hour of the incubation. Following incubation, cells were stained with fluorescently conjugated antibodies specific for mouse CD45 (eBiosciences; clone 30-F11) and for human CD45 (Invitrogen; clone HI30), CD4 (Invitrogen; clone S3.5), CD8 (BD Bioscience; clone RPA-T8), PD-1 (BioLegend; clone EH12.2H7), and an amine dye (Invitrogen) for live/dead cell discrimination. After fixation and permeabilization (BD Cytofix/Cytoperm), cells were stained with antibodies specific for IFN-γ (BD Bioscience; clone B27) and CD3 (BD Bioscience; clone SP34-2. Data were acquired on a LSRII flow cytometer (BD Biosciences) and analyzed with FlowJo software (Treestar Inc.).

In some experiments, splenocytes were cultured with JCV capsid protein VP1 peptides for 12 days and then stimulated with the same peptides for a second time for ICS. Negative control consisted of splenocytes with no peptide stimulation and positive control consisted of splenocytes stimulated with PMA. In addition, a group of splenocytes were cultured in the presence of JCV peptides but not restimulated with peptides during ICS. The IFN-γ secretions of these cells measured during the ICS were considered the baseline IFN-γ expressions. ICS result was considered positive when the percentage of IFN-γ producing CD4^+^ or CD8^+^ T cells were equal to or greater than two times the baseline value. The ICS results were reported after subtraction of baseline value as previously reported [20].

### Tetramer Staining

1×10^6^ splenocytes previously stimulated with the HLA-A*0201-restricted JCV VP1 epitopes, VP1_p36_ or VP1_p100_, were stained with a fluorescently conjugated tetramer specific to HLA-A*0201/VP1_p36_ or HLA-A*0201/VP1_p100_ for 30 minutes at room temperature and followed immediately by direct staining with antibodies specific for mouse CD45 and for human CD3, CD4, CD8, and CD45 for 25 minutes at 4°C. Prior to staining with tetramer, each sample was stained with an amine dye (Invitrogen) for live/dead cell discrimination. Data were acquired on a LSRII flow cytometer (BD Biosciences) and analyzed with FlowJo software (Treestar Inc.).

### Immunohistochemistry

Mice kidneys were extracted and fixed in 4% paraformaldehyde overnight, then placed in PBS containing 30% sucrose overnight, and finally frozen in OCT compound (Tissue tek, Sakura). Immunoperoxidase staining of anti-JCV antibody VP1 PAB597 (a generous gift from Walter Atwood) on 5 µm serial sections was performed using the Vectastain Elite ABC kit (Vector Labs) following manufacture's recommendations, and developed with DAB chromogen (Vector Labs), as previously described [21]. Sections were counter stained with Mayer's hematoxylin.

## Results

### Infection of humanized BLT mice with JCV

Similar to asymptomatic human infection with JCV, the humanized BLT mice in the experiments did not demonstrate any symptoms with exposure to JCV. Their body weights and appearances were monitored and did not differ from the phosphate buffered saline (PBS)-injected control group. Some of the mice in both study groups and in the uninfected control group suffered from graft versus host disease with symptoms of alopecia and weight loss, requiring euthanasia prior to the planned study points. However, there were no significant differences in the survival between the two virus-inoculated groups and the PBS-injected control group when analyzed using the Kaplan-Meier survival analysis (data not shown).

### Detection of JCV DNA in urine of humanized BLT mice

**Figure 1:**
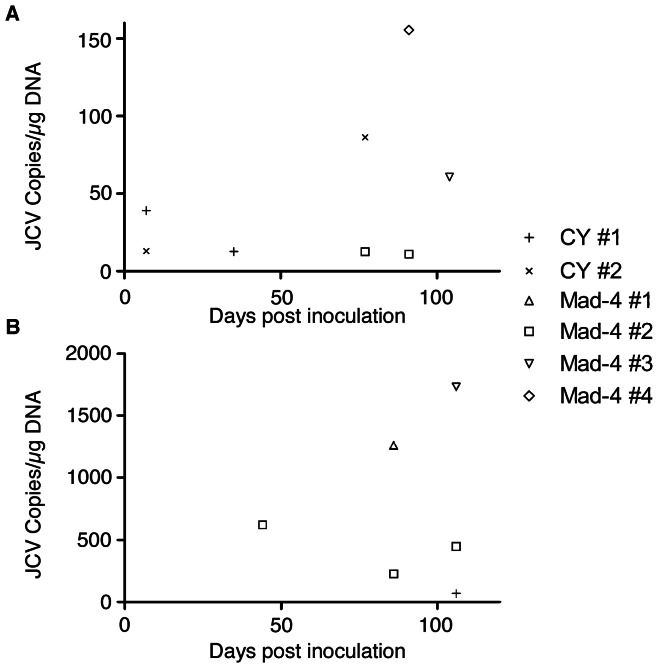
Detection of JCV DNA in the urine and blood of JCV inoculated humanized BLT mice. Only positive data are shown. (A) Urine samples were collected day 7, 22, 35, 49, 77, 91, and 104 post inoculation. JCV CY inoculation resulted in an early detection of JCV DNA in the urine on day 7 compared to first detection of JCV Mad-4 in the urine on day 77. (B) Blood samples were collected 24, 44, 65, 86, and 106 days post inoculation. Inoculation with JCV Mad-4 resulted in more frequent detection of JCV DNA in the blood compared to JCV CY. Unique symbols are used for individual mice; CY: JCV CY; Mad-4: JCV Mad-4.

To address whether humanized BLT mice inoculated with JCV can shed virus in the urine, we intraperitoneally injected 2,500 HAU (equivalent to 2.5×10^7^ viral genomes) [17] of JCV CY in 5 mice, 2,500 HAU of JCV Mad-4 in 5 mice, and 500 µl of PBS in 5 mice as negative control. We mapped the urine detection of JCV by quantitative PCR (qPCR) in urine samples collected on days 7, 22, 35, 49, 77, 91, and 104 after inoculation (**Figure 1A**). In total, 2 of 5 (40%) JCV CY-inoculated mice (CY mice) and 3 of 5 (60%) of JCV Mad-4-inoculated mice (Mad-4 mice) had detectable JCV DNA in the urine on at least one time point. In the CY mice, JCV DNA was detected in 2 mice as early as day 7. JCV DNA became undetectable in the urine of both of these mice until one had a second detection on day 35 and the other on day 77. The quantity of JCV DNA detected in urine of CY mice ranged from 13 to 86 copies per microgram of DNA. The first detection of JCV DNA in Mad-4 mice occurred in one mouse on day 77. This mouse and a second one had urine with detectable JCV DNA on day 91, and finally a third mouse had positive urine detection on day 104. The quantity of JCV DNA detected in urine of Mad-4 mice ranged from 11 to 156 copies per microgram of DNA. JCV DNA was not detected in urine from any PBS-injected control mice (PBS mice).

Presence of JCV DNA in the urine of the humanized BLT mouse inoculated with JCV parallels that of JCV infection in humans where viral shedding occurs in approximately one third of healthy asymptomatic individuals [3]. Furthermore, while viral shedding occurred in some mice on multiple occasions, no mouse was a constant shedder.

### Detection of JCV nucleic acids in blood of humanized BLT mice

To examine the presence of JCV DNA in the blood of humanized BLT mice, we sampled mice blood on days 24, 44, 65, 86, and 106 after inoculation. Altogether, 1 of 5 (20%) CY mice and 3 of 5 (60%) Mad-4 mice had detectable JCV DNA on at least one time point during the study (**Figure 1B**). JCV DNA was detected in one CY mouse on day 106 with 70 copies of JCV DNA per µg of DNA tested. JCV DNA was detected in one Mad-4 mouse on day 44, and again in the same mouse on day 106. A second Mad-4 mouse had detectable JCV DNA in blood on day 86, and a third one had detectable DNA on day 86 as well as on day 106 (**Figure 1B**). The quantity of JCV DNA detected in blood of Mad-4 mice ranged from 226 to 1730 copies per µg of DNA. Detection of JCV RNA was negative in all blood samples. No JCV DNA or RNA was detected in the blood of PBS mice.

While the detection of JCV DNA in the urine did not always correlate with detection of JCV DNA in the blood in the same mouse, some of the mice had detectable DNA in both compartments. In the CY mice, one of the two mice with detectable JCV DNA in urine early on day 7 also had detectable JCV DNA in the blood on day 106. In the Mad-4 mice, 3 mice had detectable JCV DNA in urine. Two of these mice had positive detection of JCV DNA in the blood.

These data illustrate that similar to JCV infection in humans, viral DNA can be occasionally detected in urine and peripheral blood [22]. While the mice with detectable JCV DNA in the urine did not always have detectable JCV DNA in the blood, the detection of viral DNA in both compartments in some of the mice may indicate increased viral replication or decreased immune function in these mice.

### Detection of anti-JCV antibodies in humanized BLT mice infected with JCV

**Figure 2:**
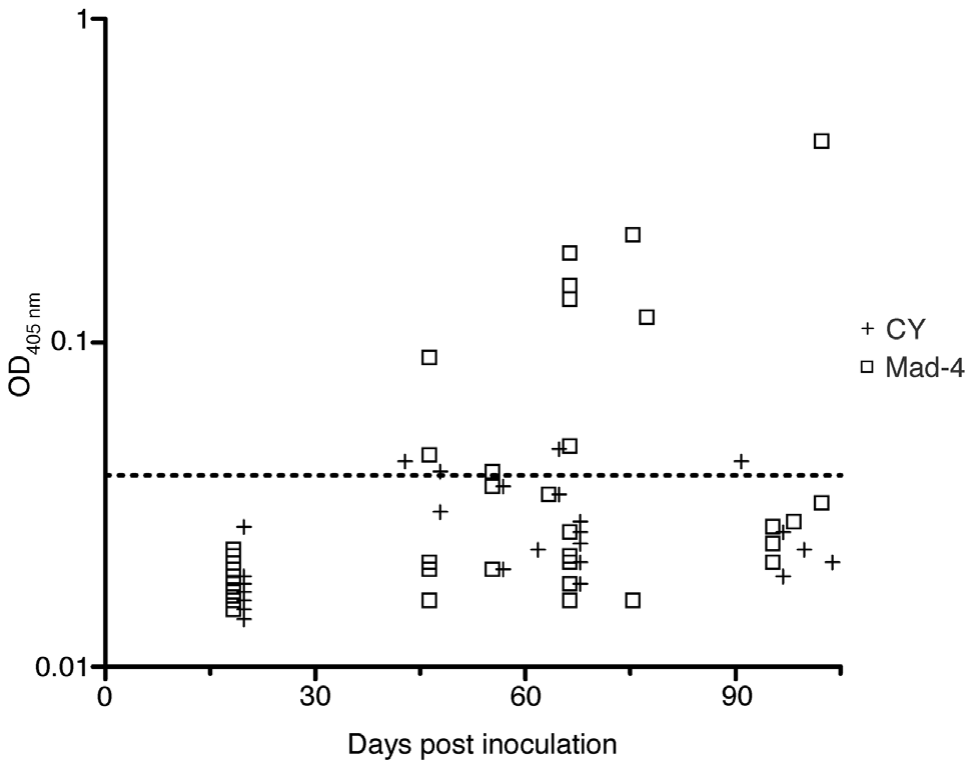
Humoral immune responses in JCV-inoculated humanized BLT mice. Anti-JCV IgM were detected 42–103 days post inoculation. JCV Mad-4 inoculation elicited a stronger humoral immune response than JCV CY inoculation. Quantitative IgM values are expressed on a logarithmic scale. Dashed line: cut-off for positive values (OD450 nm = 0.039).

Anti-JCV IgM and IgG were not detectable in mice inoculated with 2,500 HAU of the viruses on days 14–120 after infection. However, in mice inoculated with 5,000 HAU of the viruses, IgM seroconversion was detected in 4 of 13 (31%) tested mice, or 4 of 32 (12.5%) samples in the CY mice; and 7 of 16 (44%) mice, or 10 of 40 (25%) samples in the Mad-4 mice (**Figure 2**). One of 14 CY mice and 1 of 16 Mad-4 mice had only one serum available. The rest of the mice had 2 to 3 serum samples from different dates available for detection of anti-JCV IgM and IgG. In the CY mice, none of the 14 mice had detectable anti-JCV IgM on day 19. Four of the 14 mice showed IgM seroconversion on days 42, 47, 64, and 90. In the Mad-4 mice, none of the 13 mice tested had detectable anti-JCV IgM on day 19 after inoculation. Two of the 16 mice displayed IgM seroconversion on day 47, and one became IgM positive on day 56. Four other mice had IgM seroconversion on day 67, and 3 of these remained IgM positive when measured again on days 75, 78, and 103. The quantity of anti-JCV IgM in JCV Mad-4 group (median 0.127, range 0.039–0.418) was significantly higher than that of the JCV CY group (median 0.042, range 0.039–0.046), (*p* = 0.03, Mann-Whitney test).

Anti-JCV IgG was not detected in any of the animals. These serology data show that JCV-inoculated humanized BLT mice have functional human B cells that produced measurable levels of anti-JCV IgM antibodies.

### Detection of JCV-specific cellular immune response in humanized BLT mice inoculated with JCV

**Figure 3:**
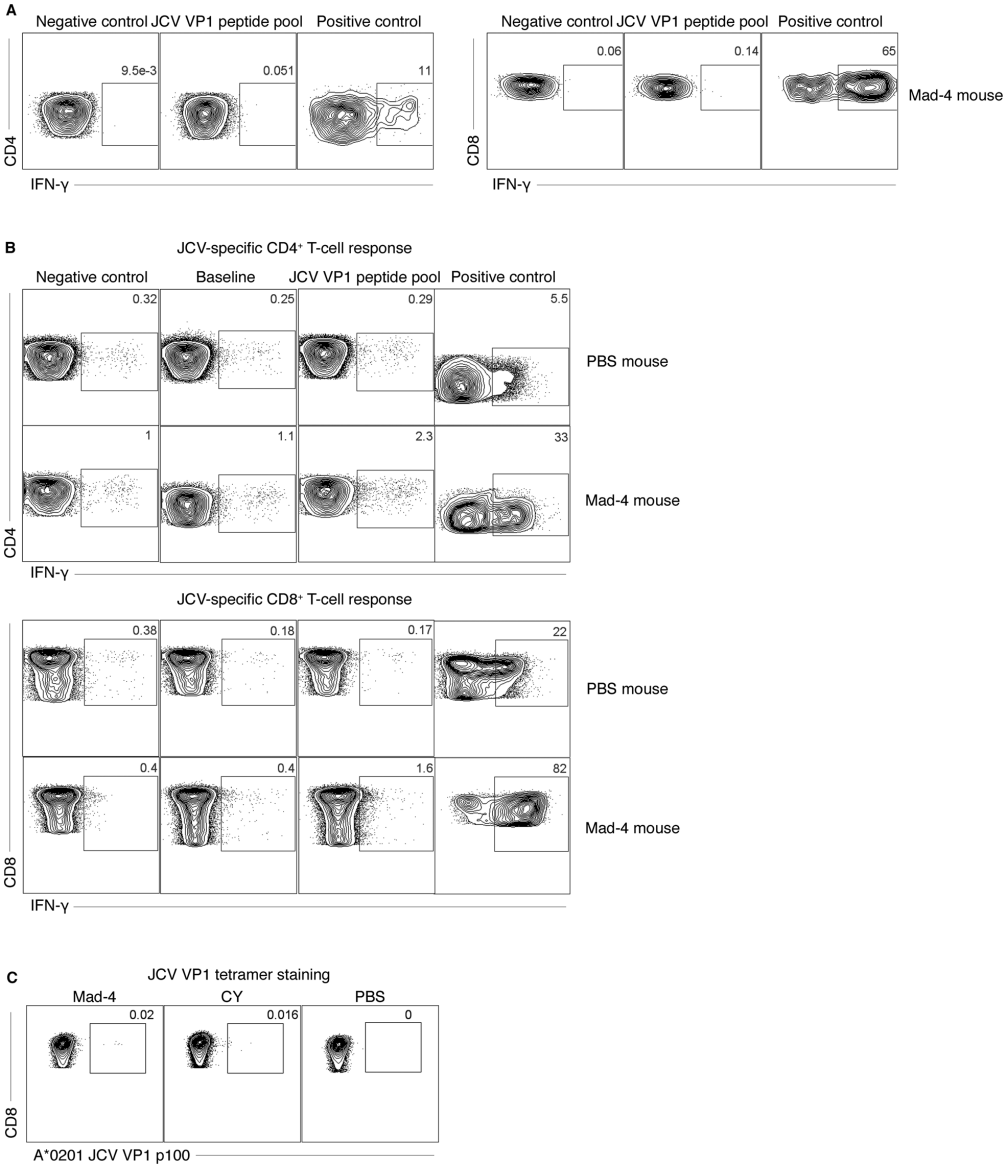
Humanized BLT mice inoculated with JCV displayed cellular immune responses against JCV VP1 antigens. (A) Intracellular staining (ICS) of splenocytes detected an increased IFN-γ expression on both CD4^+^ and CD8^+^ T cells, after stimulation with JCV peptides. (B) Culturing splenocytes with JCV capsid protein VP1 peptide pools for 12 days and then stimulated with the peptide pool a second time for ICS increased IFN-γ expression on both CD4^+^ and CD8^+^ T cells in a mouse inoculated with Mad-4, but not in a PBS-injected mouse. (C) Tetramer staining detected JCV VP1 epitope-specific CD8^+^ T cells after stimulation with A*0201-restricted JCV VP1 p100 peptide in mice inoculated with either JCV Mad-4 or CY, but not in a PBS-injected mouse. Percentages of positive cells are indicated.

Intracellular cytokine staining (ICS) for IFN-γ expression on both CD4^+^ and CD8^+^ T-lymphocytes, as well as tetramer staining for JCV-specific CD8^+^ T lymphocytes and ELISpot assays were performed using splenocytes to assess cellular immune responses against JCV in BLT mice reconstituted with HLA A*0201 positive fetal tissues. We first performed ICS with splenocytes from JCV-inoculated mice and demonstrated positive but low levels of IFN-γ expression on both CD4^+^ and CD8^+^ T cells in 3 out 4 Mad-4 mice (CD4: mean 0.003%, range 0–0.044% and CD8: mean 0.004%, range 0–0.027%) and in 4 out of 4 CY mice (CD4: mean 0.008%, range 0–0.045% and CD8: mean 0.012%, range 0–0.15%) 6 weeks after inoculation. A representative result is shown in **Figure 3A**. These low level responses are similar to those obtained in humans *ex vivo* [7], [8]. To ascertain these results, we then cultured splenocytes with JCV VP1 pooled overlapping peptides for 12 days prior to performing immune assays. A representative example of an ICS result is shown in **Figure 3B** and a tetramer staining result in **Figure 3C**. In the CY mice, 3 of 11 (27%) mice demonstrated detectable cellular immune responses with initial infection. ICS was positive in both CD4^+^ (mean: 1.1% and range 0.41–2.32%) and CD8^+^ (mean: 1.6% and range 0.41–3.73%) T cells in two different mice on day 42 and day 47 after inoculation. A third mouse had detectable JCV-specific T cells by tetramer staining on day 96.

In the Mad-4 mice, 4 of 14 (29%) mice had detectable cellular immune response to JCV with initial inoculation. One mouse demonstrated response on day 47 with detectable IFN-γ expression in both CD4^+^ and CD8^+^ T cells by ICS. A second mouse also had positive ICS detections in both CD4^+^ and CD8^+^ T cells as well as a positive ELISpot assay (194 SFU per 1×10^6^ cells, background 20) on day 91. On day 96, a third mouse had detectable JCV-specific T cells by tetramer staining as well as positive ELISpot assay (760 SFU per 1×10^6^ cells, background 180), and finally a fourth mouse had detectable IFN-γ expression in CD4^+^ T cells by ICS on day 103. The IFN-γ expression in CD4^+^ T cells by ICS had a mean of 0.60% (range: 0.24–1.36%); and on CD8^+^ T cells the mean was 0.83% (range: 0.51–1.18%).

To test whether additional exposure with the same virus can boost cellular immune response we reinoculated two CY and two Mad-4 mice on day 97 after initial inoculation with the same dose and types of virus. One mouse was assayed 40 hours after reinoculation and a second 6 days after reinoculation. In the CY mice, neither demonstrated detectable cellular immune response against JCV. In the Mad-4 mice, the first mouse sacrificed 40 hours after reinoculation showed detectable responses by both ICS (CD4^+^ T-cells: 0.26% and CD8^+^ T cells: 0.95%) and ELISpot (250 SFU per 1×10^6^ cells, background 60). The second mouse, sacrificed 6 days after reinoculation, showed detectable responses in ICS (CD4^+^ T cells: 1.58% and CD8^+^ T cells: 1.32%).

In the Mad-4 mice, there were significantly more mice with a positive cellular immune response against JCV that had detectable JCV DNA in the blood as compare to mice without cellular immune response (4/6 vs 1/10, *p* = 0.04, Fisher's Exact test).

These data demonstrate that a subset of humanized BLT mice inoculated with JCV are able to mount a cellular immune response against JCV, as is the case in humans. Presence of JCV DNA in the blood increased the likelihood of detection of a cellular immune response against JCV in the Mad-4 mice. The immune responses data are summarized in **Table 1**.

**Table 1:** Detection of anti-JCV immune response in JCV-inoculated humanized mouse.

		Days post infection at Sacrifice	Serology	ICS		Tetramer	ELISpot
Virus	ID#		IgM	CD4	CD8		
**JCV CY**	1	42	+	+	+	−	−
	2	47	−	+	+	−	−
	3	47	+	−	−	−	−
	4	53	N/A	N/A	N/A	N/A	N/A
	5	56	−	−	−	−	N/A
	6	56	−	−	−	−	N/A
	7	61	−	−	−	−	−
	8	64	+	−	−	−	−
	9	64	−	−	−	−	−
	10	90	+	−	−	−	−
	11	96	−	−	−	+	−
	12	96	−	−	−	−	−
	13^A^	99	−	−	−	−	−
	14^B^	103	−	−	−	−	−
**JCV Mad-4**	15	47	+	−	−	−	−
	16	47	−	−	−	−	−
	17	47	+	+	+	−	−
	18	56	−	−	−	−	N/A
	19	56	+	−	−	−	−
	20	56	−	−	−	−	N/A
	21	64	−	−	−	−	−
	22	70	−	−	−	−	−
	23	75	+	−	−	−	−
	24	78	+	−	−	−	−
	25	91	−	+	+	−	+
	26^A^	94	−	+	+	−	+
	27	96	−C	−	−	+	+
	28	96	−	−	−	−	−
	29	103	+	+	−	−	−
	30^B^	103	−	+	+	−	−

A Mouse reinoculated with virus then sacrificed 40 hours after reinoculation.

B Mouse reinoculated with virus then sacrificed 6 days after reinoculation.

C IgM detected on day 67, but reverted to negative at sacrifice 96 days after inoculation.

### Analysis of T-lymphocytes exhaustion in JCV infected humanized BLT mice

**Figure 4:**
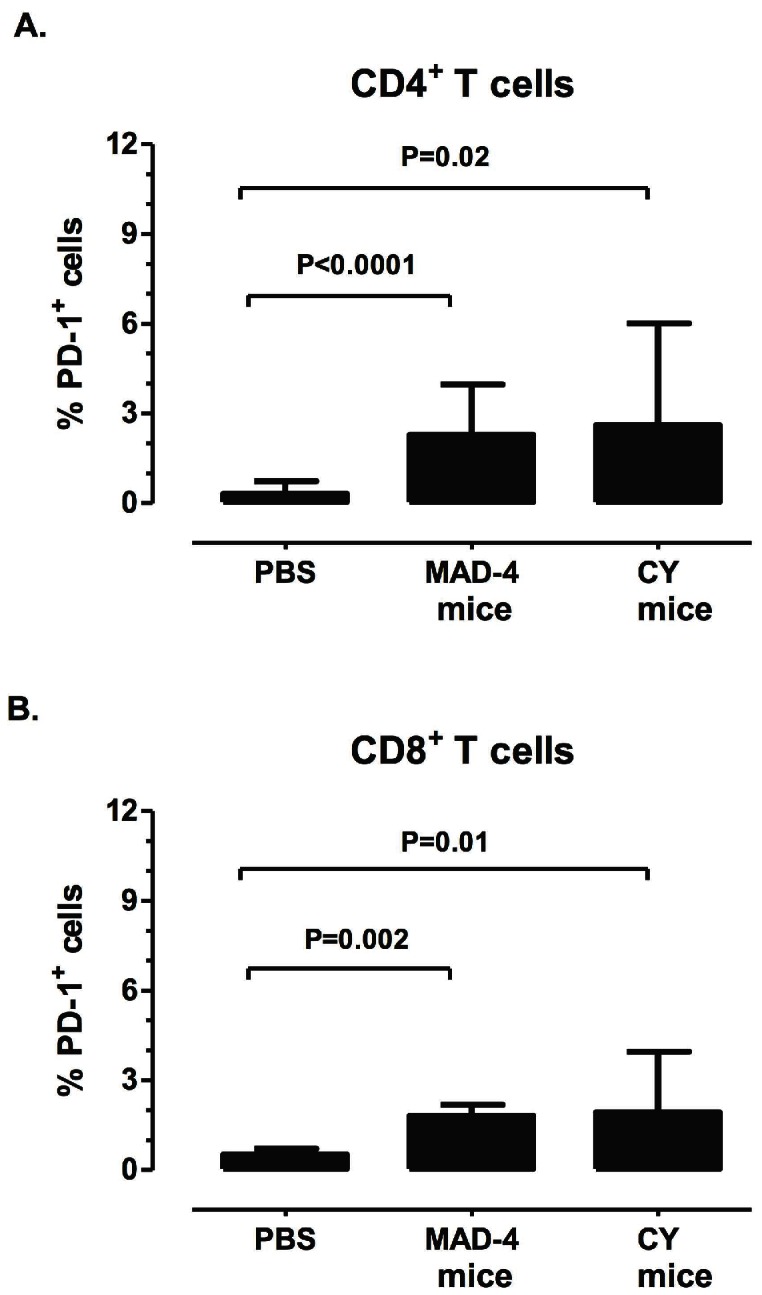
JCV-inoculated humanized BLT mice showed increased expression of the cell exhaustion marker, PD-1, on splenocytes. PD-1 expression was measured on splenocytes after stimulation with JCV VP1 peptide pools. A significantly higher percentage of CD4^+^ and CD8^+^ T cells expressed PD-1 in either the JCV Mad-4 or CY mice as compared to the PBS mice. Bars illustrate the means and standard deviation above the means in each group.

We examined the immune exhaustion marker, programmed cell death (PD-1), expression in humanized BLT mice inoculated with JCV CY and Mad-4. Six weeks after inoculation, both the CY and Mad-4 mice had significant increase in PD-1 expressions on CD4^+^ T cells, compared to the PBS mice (*p* = 0.02 and <0.0001) (**Figure 4A**). This increase was also observed in the CD8^+^ T cells when comparing both the CY and Mad 4 mice to the PBS mice (*p* = 0.01 and 0.002) (**Figure 4B**). We have previously shown that, PD-1 was elevated on both CD4^+^ T cells and CD8^+^JCV-specific T cells in patients with PML [23]. The up regulation of PD-1 in the virus-inoculated mice suggests that JCV infection may have a similar effect on immune exhaustion in the mouse model.

### Detection of JCV in kidney of humanized BLT mice

**Figure 5:**
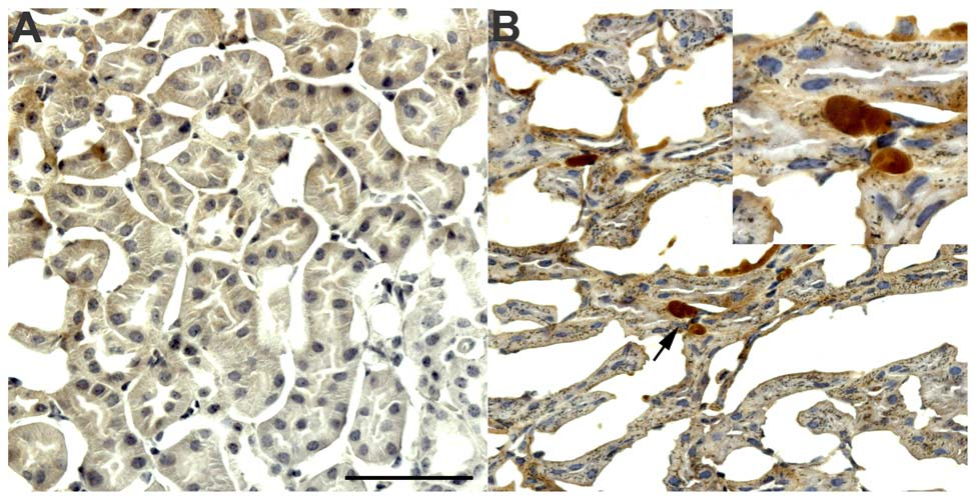
JCV-inoculated humanized BLT mice showed rare detection of JCV VP1 protein in the kidney. Immunohistochemistry staining of JCV VP1 protein was not detected in the kidney of PBS-inoculated mice (A), but was detected in rare kidney cells in JCV Mad-4 inoculated mice (B). The images are magnified 40-fold, and the inset is magnified 100-fold. Scale bar = 100 µm.

To determine JC viral tissue tropism in this model, we analyzed the organs of JCV-inoculated humanized BLT mice for the presence of JCV DNA. While no JCV DNA was detected in tissues from brain, intestine, liver, lung, lymph nodes, or bone marrow, we consistently detected JCV DNA in the kidney of Mad-4 mice. In humanized BLT mice inoculated with 2,500 HAU of either JCV Mad-4 or CY virus, JCV DNA was detected in 4 of 9 (44%) Mad-4 mice and in none of the CY mice 120 days after inoculation. In humanized BLT mice inoculated with 5,000 HAU of either JCV Mad-4 or CY virus, JCV DNA was detected in the kidney from 9 of 16 (56%) Mad-4 mice and in 7 of 12 (58%) CY mice, harvested days 42 to 103 after inoculation. Kidney tissues from PBS mice did not have detectable JCV DNA. The quantities of JCV DNA in the kidney tissues were determined by qPCR and were all below 500 copies/µg of DNA. As a confirmatory experiment, we amplified, cloned, and sequenced the JCV RR from the kidneys of multiple Mad-4 mice. Sequencing results confirmed that the RR is same as the JCV Mad-4 virus used for inoculation (data not shown). Furthermore, we performed immunohistochemistry staining and detected rare kidney tubular cells containing JCV VP1 protein in JCV Mad-4-inoculated mice (**Figure 5**).

## Discussion

Our pilot data demonstrate that the humanized BLT mouse may be useful for the study of immune response against JCV. Similar to humans, primary infection with JCV did not elicit any specific symptoms. Since infection with JCV is asymptomatic, it is difficult to calculate the optimal dose of viral inoculum. While mice inoculated with 2,500 HAU of either JCV Mad-4 or CY did not produce detectable anti-JCV IgM or IgG within 120 days after initial inoculation, IgM seroconversion was detected in 29% of CY and 44% of Mad-4 mice inoculated with 5,000 HAU of the virus. The absence of IgG may be caused by the fact that the reconstituted immune system in this model is immature and longer exposure to virus is necessary to trigger IgG production, as seen with HIV [10]. Thus, this BLT mouse model may more accurately depict JCV immune response by human fetus. Furthermore, we injected JCV intraperitoneally based on our experience that intraperitoneal injection of HIV achieved similar viral infection as intravenous injection in BLT mice. However, this may be different for JCV. Thus, higher quantities, longer exposure to JCV, and intravenous injection can be tested in future experiments to elicit possibly stronger immune responses.

JCV was occasionally detected in the blood of virus-inoculated humanized BLT mice. Similar to human infection, JCV is occasionally detected in the blood in 20–60% of the mouse samples, up to 106 days post inoculation, suggesting persistent low level viral replication. Alternatively, JCV DNA detections may result from residual inoculums; this possibility is unlikely due to the elapsed time post inoculation. While JCV DNA is usually not detected in the peripheral blood of healthy individuals, it was detected in 13% of PBMC and in 22% of plasma from HIV positive individuals without PML [22]. The detection of JCV DNA in the plasma of these mice may be due to incomplete immune control of JCV and perhaps indicate that the mice immune system is more comparable to those of humans with immunosuppressions.

JCV was detected in the urine of virus-inoculated mice at a frequency of 40%–60% during the course of our experiments as compared to detection in one third of healthy adults as well as immunosuppressed patients with or without PML. JCV CY virus was detected in the urine as early as 7 days after infection, prior to detection in the plasma and prior to the detection of JCV Mad-4 virus. This may indicate that JCV CY is preferentially processed through the renal system. While it is not known if JCV CY or Mad-type of virus is responsible for primary infection in humans, JCV CY is the predominant virus detected in urine and in kidneys in both healthy individuals as well as PML patients. Therefore, these data provide further evidence that the presence of JCV in the renal system may be partially determined by the regulatory region of the virus. The exact mechanisms of regulatory region exerting influence on this selection need to be further investigated.

Similar to other polyomaviruses, JCV is species-specific and can only replicate in humans. However, while the humanized BLT mice in our experiments had no human kidney tissues, JCV DNA and protein were detected in the mouse kidney tissues as late as 103 days after inoculation. It is possible that mouse kidney epithelial cells express receptors similar to those in humans, facilitating JC virus entry. Further investigation is needed to characterize the viral entry and determine whether JCV enters the kidneys as free virus or attached to cells such as lymphocytes. Once inside the kidney epithelial cells, it is unclear if mice cellular machinery can support JCV replication though previous studies suggest that the rodent cellular machinery would not support productive infection [24]. The detection of JCV in the urine could be an indication of active replication or could be due to the release of viral DNA into the lumen of the tubules after death of the kidney epithelial cells. While about half of the kidneys contained detectable JCV DNA, fewer urine samples contained JCV DNA. This may indicate that JCV can be latent in the kidney of BLT mice without active replication and release into the urine. The detection in urine increased with increased dose of inoculated JCV. Therefore, a higher JCV inoculum may further increase urine detection of JCV in this model.

We and others have previously studied cellular immune responses to JCV in humans and showed that the major limitation to immune response assays is the low frequency of detectable JCV specific T-cells in peripheral blood *ex vivo* [7], [8]. Therefore, to better characterize the JCV-specific cellular immune response and to compare with human JCV immune responses, we performed ICS, tetramer staining, and ELISpot after stimulation of mice splenocytes with JCV peptides *in vitro*, similar to our previous experimental methods with human blood samples [20]. To control for the culturing conditions, we have developed stringent criteria to evaluate assay results in order to account for false positives that may have risen from *in vitro* experimentation. The magnitude of the immune responses measured in our model is comparable to those seen in healthy individuals. However, humoral or cellular immune responses were detected only in a subgroup of mice in our study. This may be due to several factors. First, we performed immune assays starting day 42 after inoculation. Therefore, some mice may have generated earlier responses which were no longer detectable at that time point. Second, although the JCV was latent in the kidneys, the lack of robust active viral replication may have also reduced the immune response over the long term. Transplanting human kidney tissues in these BLT mice may provide a source for constant JC viral replication and increase immune response. Third, higher quantity of JCV inoculum may also elicit increased magnitudes of response in a larger number of animals. Future efforts should be directed toward boosting this immune response. Lastly, while human immune responses to JCV are well studied in adults, little is known of the magnitude or dynamics of JCV-specific immune responses in primary infection, which may be represented by this model.

Our data suggest several virological differences between JCV CY and Mad-4. Humanized BLT mice inoculated with JCV CY and Mad-4 virus showed differences in viral DNA load and the magnitude of the immune response. This may be due to several factors. First, while the same dose of JCV CY and Mad-4 were given to the mice, JCV CY may have a slower rate of viral replication in the humanized BLT mice, resulting in quantitatively fewer antigens. Our finding that JCV DNA was detected at a higher frequency in the blood of Mad-4 mice as compared to CY mice (60% vs. 20%) also supports this argument. Second, these two viruses may have different interactions with the host immune cells. In addition, mice infected with JCV Mad-4 produced significantly more anti-JCV IgM antibodies than mice infected with JCV CY. Lastly, JCV CY and Mad-4 may induce different immune memory and rate of response. Whereas the cellular immune response in Mad-4 mice is correlated with the detection of JCV DNA in peripheral blood, this was not seen in CY mice. The immune response differences are further demonstrated by reinoculation of two mice from each group. While both of the Mad-4 reinoculated mice showed detectable cellular immune responses, this was not the case with the CY reinoculated mice. These differences in both humoral and cellular immune responses to these two viruses argue against a mere reaction to the initial viral inoculums and suggest a low level active infection.

Elucidation of JC virology is necessary in order to better understand JCV pathogenesis and PML. These results are the first *in vivo* demonstration of distinct virological and immunological differences between JCV Mad-4 and CY. The quantitative differences in DNA viral load detected in blood and urine may be due to different replication efficiency associated with sequence differences in the non-coding RR of these viruses. These differences in the RR can alter the binding of nuclear transcription factors. The host immune response differences may be due to the amino acid differences between JCV CY and Mad-4. These viral strains have a total of 17 amino acid differences in the six JCV proteins, including seven located in the capsid proteins VP1, -2 and -3. Amino acid changes in the VP1 protein have been described in JCV strains isolated from the brains of PML patients compared to those found in the urine of healthy people [25], [26],[27]. Further studies using the humanized BLT mouse model will be needed to determine the role of the immune response in the differential containment of JCV Mad-4 and CY.

Although the detected levels of JC viral DNA and JCV-specific immune responses are low in our study, our data suggests that a subgroup of the JCV-inoculated mice were indeed infected with the virus. First, we were able to detect JCV DNA in the urine and blood of some inoculated mice as late as 104 days after inoculation. Second, in a subgroup of the virus-inoculated mice, we demonstrate positive cellular and humoral immune responses using the stringent criteria used for human responses. Lastly, the lymphocyte exhaustion marker, PD-1, associated with JCV infection in humans, is also elevated on both CD4^+^ and CD8^+^ T cells in virus-inoculated mice.

While our humanized BLT mouse model does not recapitulate the disease PML, our data showed that this model may be useful in studying the immune response of a human-specific virus, such as JCV. Using humanized BLT mice, we have demonstrated positive cellular and humoral immune responses, albeit at low levels. Better understanding of viral interactions with host immune response is crucially needed to prevent PML in immunosuppressed patients. The current lack of an animal model greatly hampers JCV research. Therefore, this humanized BLT mouse model can be utilized in future studies in deciphering the immune response in JCV and HIV co infection, and chemical immunosuppression with monoclonal antibodies.

## Conclusions

The humanized BLT mice provide a possible animal model of human immune response to JCV. The human lymphocytes engrafted in the mouse model generated both humoral and cellular responses against JCV. Furthermore, we have demonstrated distinct virological and immunological differences between JCV CY and JCV Mad-4. This model may prove very valuable for studying JCV host immune responses and aid in elucidating alterations caused by immuno modulations, and devise better therapeutic interventions.
